# The Ligand Binding Domain of GCNF Is Not Required for Repression of Pluripotency Genes in Mouse Fetal Ovarian Germ Cells

**DOI:** 10.1371/journal.pone.0066062

**Published:** 2013-06-07

**Authors:** Leah M. Okumura, Bluma J. Lesch, David C. Page

**Affiliations:** 1 Howard Hughes Medical Institute, Chevy Chase, Maryland, United States of America; 2 Whitehead Institute, Cambridge, Massachusetts, United States of America; 3 Department of Biology, Massachusetts Institute of Technology, Cambridge, Massachusetts, United States of America; University Paris Diderot/University Paris 7, France

## Abstract

In mice, successful development and reproduction require that all cells, including germ cells, transition from a pluripotent to a differentiated state. This transition is associated with silencing of the pluripotency genes *Oct4* and *Nanog*. Interestingly, these genes are repressed at different developmental timepoints in germ and somatic cells. Ovarian germ cells maintain their expression until about embryonic day (E) 14.5, whereas somatic cells silence them much earlier, at about E8.0. In both somatic cells and embryonic stem cells, silencing of *Oct4* and *Nanog* requires the nuclear receptor GCNF. However, expression of the *Gcnf* gene has not been investigated in fetal ovarian germ cells, and whether it is required for silencing *Oct4* and *Nanog* in that context is not known. Here we demonstrate that *Gcnf* is expressed in fetal ovarian germ cells, peaking at E14.5, when *Oct4* and *Nanog* are silenced. However, conditional ablation of the ligand-binding domain of *Gcnf* using a ubiquitous, tamoxifen-inducible Cre indicates that *Gcnf* is not required for the down-regulation of pluripotency genes in fetal ovarian germ cells, nor is it required for initiation of meiosis and oogenesis. These results suggest that the silencing of *Oct4* and *Nanog* in germ cells occurs via a different mechanism from that operating in somatic cells during gastrulation.

## Introduction

In mice, several factors are essential for maintaining pluripotency in the early embryo and in embryonic stem (ES) cells, including the POU family transcription factor OCT4 (also called POU5F1) and the homeobox protein NANOG. *Oct4* is expressed in the inner cell mass (ICM) of the blastocyst. In its absence, ICM cells differentiate into trophectoderm-like cells and embryos are inviable [1]. *Nanog* is expressed in the epiblast when it differentiates from the hypoblast, and functions to maintain pluripotency in the early embryo. *Nanog*-deficient embryos fail to form an epiblast and die shortly after implantation [2].

Both *Oct4* and *Nanog* are down-regulated in somatic cells during gastrulation, but they continue to be expressed in primordial germ cells (PGCs), along with several other pluripotency genes, including *Sox2* and *Dppa3* (also known as *Stella*) [3], [4]. Pluripotency gene expression is maintained in germ cells until after they migrate to the developing gonads, which they enter by E11.5 [5], [6]. In particular, *Oct4* and *Nanog* are both required for survival of PGCs. Conditional ablation of *Oct4* or *Nanog* specifically in PGCs results in apoptosis [7], [8].

In XX (female) embryos, germ cell expression of pluripotency genes is maintained until about E14.5, when the germ cells enter meiosis [5], [8]. However, it is not known whether this repression of pluripotency genes in fetal ovarian germ cells is required for meiotic initiation. In addition, the mechanism of repression of pluripotency genes in fetal germ cells is largely unknown. Recent studies in C57BL/6 mice demonstrated that silencing of pluripotency genes in fetal germ cells depends on the gene *Dazl* (*Deleted in azoospermia-like*), which encodes an RNA-binding protein expressed specifically in germ cells; *Dazl*-deficient germ cells display extended expression of pluripotency markers [9]. However, as *Dazl* regulates a broad transition in fetal germ cell state, that of PGCs into gametogenesis-competent cells (GCCs) [9], its role in down-regulating pluripotency genes may be indirect.

We set out to determine what factors might directly mediate the effects of *Dazl* in repressing the pluripotency genes *Oct4* and *Nanog* in fetal ovarian germ cells. GCNF (germ cell nuclear factor, Nr6a1) was a likely candidate: it is an orphan nuclear receptor known to repress *Oct4* and *Nanog* in the soma during gastrulation [10], [11]. The GCNF protein binds directly to DR0 sites in the promoters of both the *Oct4* and *Nanog* genes and is thought to repress them via interaction with the nuclear corepressors SMRT and NCoR [10], [11], [12]. We hypothesized that GCNF might also be required for repression of *Oct4* and *Nanog* in fetal ovarian germ cells.

Using high-throughput mRNA sequencing and single molecule fluorescence *in situ* hybridization, we found that, in fetal ovarian germ cells, *Gcnf* expression peaks at the time when *Oct4* and *Nanog* are repressed. To explore the function of *Gcnf* in fetal ovarian germ cells, we generated whole-body and conditional-knockout mice in which the ligand-binding domain (LBD) of GCNF was disrupted. We found that, although disruption of the LBD of GCNF prevented *Oct4* repression in the soma during gastrulation, it did not affect *Oct4* or *Nanog* repression in fetal ovarian germ cells. In *Gcnf*-mutant embryos, all aspects of germ cell development examined were similar to those found in wild-type embryos. We conclude that *Gcnf* is not required for fetal ovarian germ cell development, and that repression of pluripotency genes must be differentially regulated in somatic and germ cells.

## Results

### Expression of *Gcnf* in fetal ovarian germ cells

We first determined if *Gcnf* expression coincided with the down-regulation of *Nanog* and *Oct4* in fetal ovarian germ cells. Two different approaches were used: analysis of high-throughput mRNA sequencing data and single molecule fluorescence *in situ* hybridization (smFISH). Both NANOG and OCT4 proteins are detectable at E13.5, are down-regulated at E14.5, and are undetectable by E16.5 [5], [13]. If *Gcnf* is required for this down-regulation, it should be expressed in germ cells at the time when *Nanog* and *Oct4* repression occurs.

**Figure 1:**
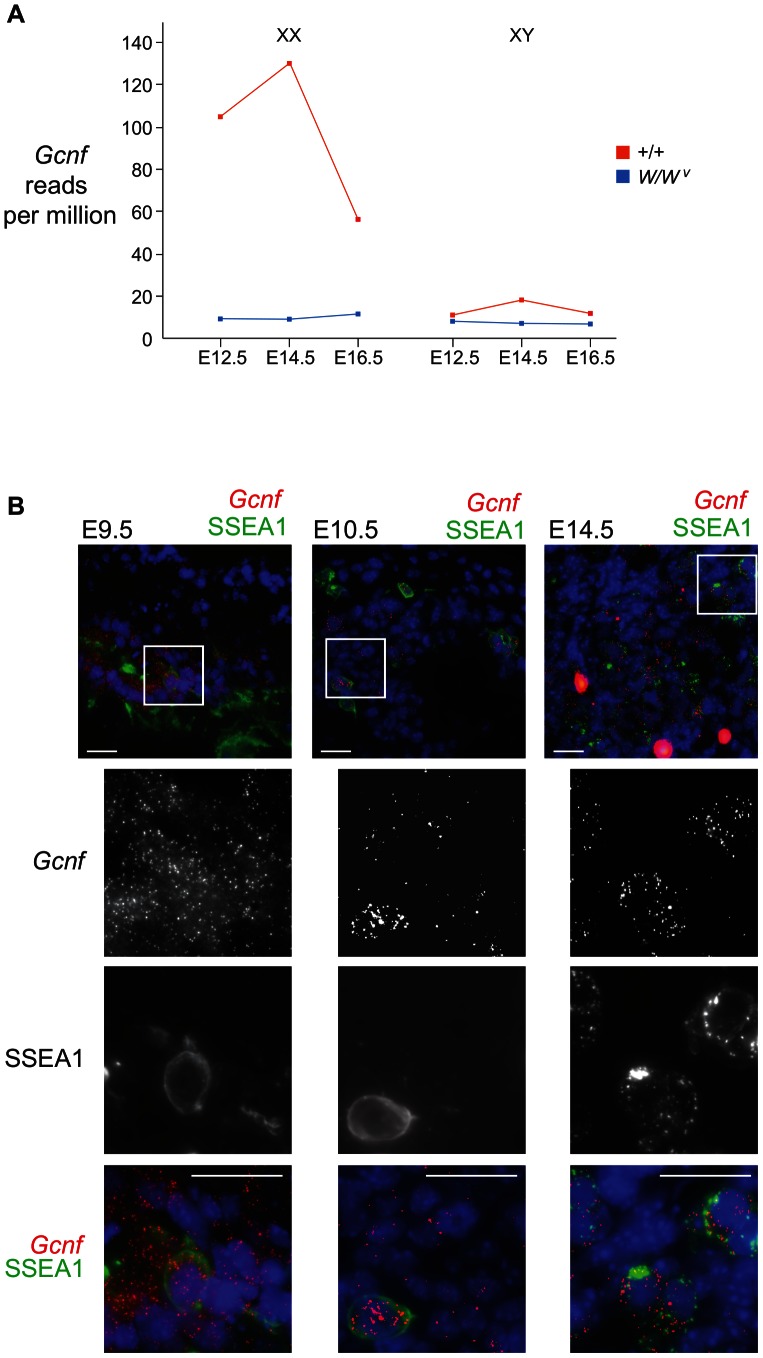
*Gcnf* expression in fetal ovarian germ cells. (A) Levels of *Gcnf* transcript in female (XX) and male (XY) gonads from wild-type and germ-cell-depleted (*W/W^v^*) mutant embryos at E12.5, E14.5, and E16.5, as determined by Illumina sequencing of gonadal RNA. Plotted here are average numbers of reads of *Gcnf* per million total reads from two individual biological replicates. (B) Single molecule fluorescence in situ hybridization for *Gcnf* mRNA (red) in sections of XX genital ridges or gonads, with germ cells marked by SSEA1 (green), and nuclei marked by DAPI staining (blue). Large red-orange spots in the E14.5 image are auto-fluorescent blood cells. Boxes indicate areas shown in higher magnification below each image. Scale bar, 20 um.

First, we analyzed high-throughput mRNA sequencing data from wild-type gonads as well as gonads lacking germ cells (derived from *Kit^W^/Kit^W-v^* mutant mice), at E12.5, E14.5, and E16.5 (data kindly provided by Jacob Mueller and Mark Gill). We found that *Gcnf* transcripts are present in wild-type fetal ovaries as early as E12.5, with peak expression at E14.5, and a precipitous drop in expression at E16.5 (Fig. 1A). *Gcnf* transcript levels are very low at all three time points in wild-type fetal testes as well as in germ-cell-deficient *W/W^v^* gonads of both sexes (Fig. 1A).

Second, we used smFISH to examine *Gcnf* transcripts in wild-type mesentery, genital ridges, or gonads at E9.5, E10.5, and E14.5 (Fig. 1B). Germ cells were identified by staining for the germ-cell-specific cell-surface antigen SSEA1, as well as by their distinctive nuclear morphology, visualized by DAPI staining. We found that, at E9.5, consistent with published data [14], *Gcnf* transcripts were widespread and easily detectable in both somatic and germ cells. By contrast, at E10.5, most cells did not express *Gcnf*; its mRNA could be detected in only scattered somatic and germ cells. By E14.5, mRNA expression of *Gcnf* had increased dramatically in germ cells, but no expression was detected in somatic cells. Taken together, these results indicate that at the time when pluripotency markers are repressed in the fetal ovarian germ cells, *Gcnf* is expressed specifically in these cells.

### Generation and phenotypic characterization of mutant mice lacking the ligand-binding domain of *Gcnf*

**Figure 2:**
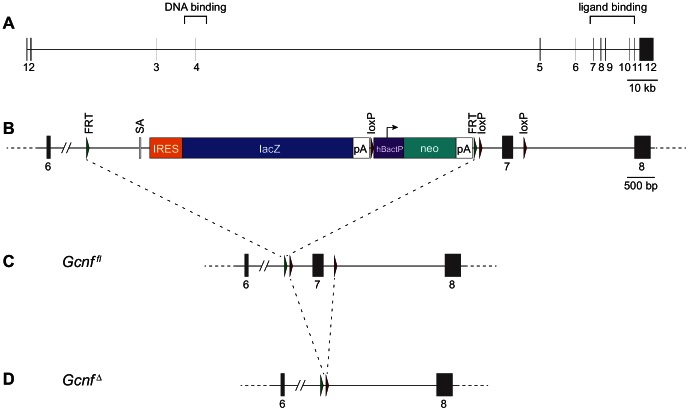
Targeted disruption of *Gcnf*. (A) Genomic structure of wild-type *Gcnf* locus. Exons 1–12 are numbered and shown as gray vertical lines or boxes. The exons encoding the DNA binding domain (previously disrupted by Chung, et al. [15]) and ligand binding domain (disrupted here) are indicated. (B) Gene-trap allele (*Gcnf^gt^*) present in ES cells obtained from EUCOMM (only exons 6–8 are shown). Gene-trap cassette contains a splice acceptor (SA) and internal ribosome entry site (IRES) upstream of a lacZ reporter gene followed by a polyadenylation (pA) signal. Selection cassette consists of a neomycin resistance gene (neo) driven by an autonomous promoter (hBactP) and pA signal. Both cassettes are flanked by FLP-recombination target (FRT) sites. (C) After FLP-mediated recombination *in vivo*, the gene-trap and neomycin-resistance cassettes are removed, leaving a true conditional knockout allele, referred to as *Gcnf^fl^*, with loxP sites flanking exon 7. (D) Tamoxifen administration induces nuclear localization of Ubc-Cre-ERT2, resulting in Cre-mediated recombination between the loxP sites, which deletes exon 7. We refer to this allele as *Gcnf^Δ^*. All structures drawn to scale; scale bars for (A) and (B–D) are shown.

To determine whether *Gcnf* is required for the silencing of *Oct4* and *Nanog* in fetal ovarian germ cells, we generated *Gcnf*-mutant mice from targeted ES cells in which the genomic region encoding the LBD of GCNF is disrupted. We obtained targeted ES cells from the EUCOMM (European Conditional Mouse Mutagenesis) Project. This targeted allele, referred to here as *Gcnf^gt^*, contains a gene trap cassette and loxP sites flanking exon 7 (Fig. 2A,B). Exon 7 encodes the N-terminal portion of the LBD, which has been shown to be important for interaction of GCNF with the corepressors NCoR and SMRT [11], [12].

We first tested whether disruption of the LBD of GCNF resulted in defects similar to those observed when the DNA binding domain (DBD) is disrupted. Previously, disruption of the DBD has been shown to abrogate GCNF function, resulting in severe morphological defects by E9.5, including failure to turn from the lordotic to the fetal position, failure of the neural tube to close completely, a truncation in somite development, and the failure to downregulate *Oct4* expression throughout the embryo. DBD-mutant embryos eventually die, surviving only until about E10.5 [11], [15], [16].

We tested LBD-mutants for prolonged expression of *Oct4* and morphological defects at E9.5. Mice carrying the *Gcnf^gt^* allele were first bred to mice carrying a ubiquitously expressed FLP recombinase, *ACTB:FLPe* [17], in order to excise the gene trap and NeoR cassette. This left behind a single FRT site and LoxP sites flanking exon 7, enabling conditional deletion of exon 7 (Fig. 2C). This allele is referred to as *Gcnf^fl^*. We then generated mice carrying both the *Gcnf^fl^* allele and the ubiquitously-expressed tamoxifen-inducible *Ubc-Cre-ERT2* [18], and injected adult *Gcnf^fl/+^; Ubc-Cre-ERT2* males with tamoxifen to obtain adult male mice heterozygous for a full-body deletion of *Gcnf* exon 7. Deletion of exon 7 should cause a frameshift and a premature stop codon near the 5′ end of exon 8, thereby disrupting translation of the LBD (Figure 2C,D). We refer to this allele as *Gcnf^Δ^*. We crossed the *Gcnf^Δ/+^* founder males obtained in this manner with wild-type females to propagate the *Gcnf^Δ^* allele.

**Figure 3:**
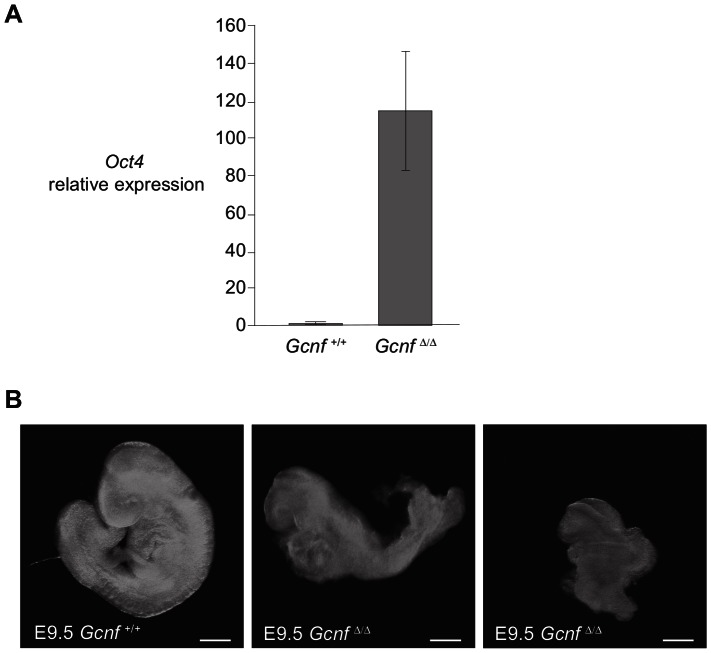
*Gcnf^Δ/Δ^* mutants fail to down-regulate *Oct4* and display morphological defects. (A) Quantitative RT-PCR for *Oct4* on E9.5 embryos. Plotted here are average fold changes (relative to *Gcnf^+/+^* whole embryo; all values normalized to *Actb*) of at least four independent biological replicates. Error bars show standard deviations among biological replicates. (B) Images of *Gcnf^+/+^* wild-type and *Gcnf^Δ/Δ^* mutant embryos at E9.5. All embryos are oriented with head facing left. Scale bar, 1 mm.

We examined mutant embryos homozygous for the full-body *Gcnf* deletion allele (*Gcnf^Δ/Δ^*) as well as wild-type (*Gcnf^+/+^*) and heterozygous (*Gcnf^Δ/+^*) littermate controls at E9.5. As expected, quantitative RT-PCR on whole embryos at E9.5 showed that wild-type *Gcnf^+/+^* controls expressed low levels of *Oct4* transcript at this time point. In comparison, *Gcnf^Δ/Δ^* homozygotes had greatly increased levels of *Oct4* expression, suggesting that the LBD of GCNF is required for the repression of *Oct4* during gastrulation (Fig. 3A). Additionally, at E9.5, *Gcnf^Δ/Δ^* homozygotes lacking the LBD displayed severe morphological defects similar to those previously reported in mutants lacking the DBD [11], [15]. *Gcnf^Δ/Δ^* homozygotes were smaller than their wild-type littermates, had truncated somitogenesis, and failed to turn from the lordotic to the fetal position (Fig. 3B).

Because homozygotes lacking the LBD of GCNF recapitulate the phenotype of *Gcnf* mutants lacking the DBD, we conclude that the LBD is required for GCNF function during early embryogenesis.

### Conditional deletion of *Gcnf* ligand-binding domain

Ovarian germ cells down-regulate expression of pluripotency markers at about E14.5 [5], [13]. We next wished to determine whether *Gcnf* is required for the repression of *Oct4* and *Nanog* in ovarian germ cells at this time. Because *Gcnf* is required for embryonic development, and full-body mutants are inviable beyond E10.5 [15], [16], we generated conditional knockout mice.

To generate embryos conditionally lacking exon 7, we mated heterozygous *Gcnf^fl/+^* females to heterozygous *Gcnf^fl/+^* males carrying the ubiquitously expressed tamoxifen-inducible *Ubc-Cre-ERT2*. Pregnant females were injected with tamoxifen twice, at E10.5 and E11.5, to induce Cre-mediated recombination throughout the embryo. This time interval was chosen for Cre induction because it is after *Gcnf* is down-regulated throughout the embryonic soma [14] but before *Gcnf* is up-regulated in ovarian germ cells at E12.5.

**Figure 4:**
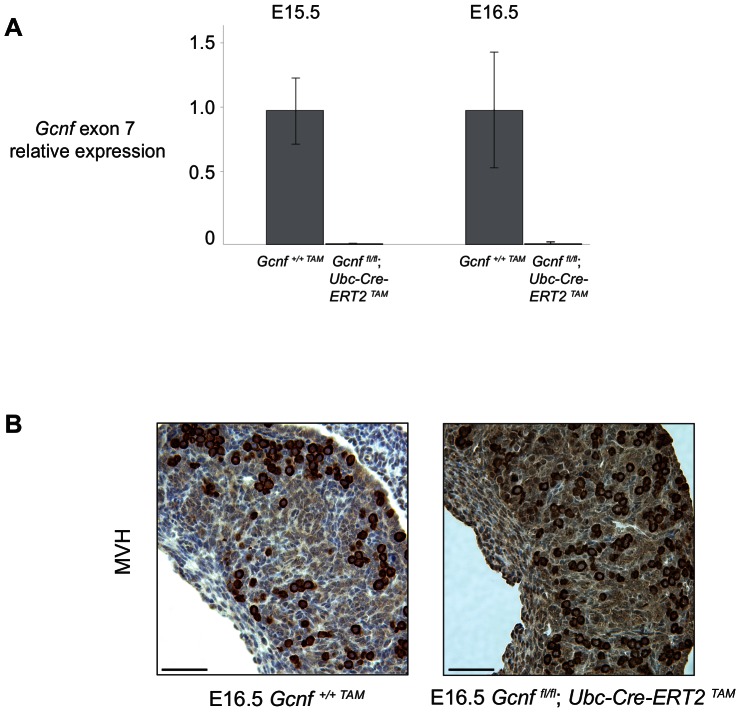
Exon 7 of *Gcnf* is efficiently deleted in mutants. (A) Quantitative RT-PCR for exon 7 of *Gcnf* in ovaries of *Gcnf^fl/fl^; Ubc-Cre-ERT2^TAM^* mutants and *Gcnf^+/+TAM^* wild-type littermate controls at both E15.5 and E16.5. Plotted here are average fold changes (relative to *Gcnf^+/+TAM^* ovaries; all values normalized to *Hprt*) for two (at E15.5) or three (at E16.5) independent biological replicates. Error bars show standard deviations among biological replicates. (B) Immunostaining for germ cell marker MVH in *Gcnf^+/+TAM^* wild-type and *Gcnf^fl/fl^; Ubc-Cre-ERT2^TAM^* mutant ovaries at E16.5. Anterior is to the left. Scale bar, 50 um.

To determine the efficiency of recombination in the fetal ovary, we collected ovaries from tamoxifen-treated mutant embryos (*Gcnf^fl/fl^; Ubc-Cre-ERT2^TAM^*) and tamoxifen-treated wild-type littermate controls (*Gcnf^+/+TAM^*) at E15.5 and E16.5 and used quantitative RT-PCR (qRT-PCR) to measure levels of *Gcnf* exon 7-containing transcript. In all mutants examined, recombination was nearly complete, with an average of less than 1.5% of exon 7 transcript detectable compared to controls (Fig. 4A).

To ensure that the inability to detect exon 7-containing transcript was not due to an absence of germ cells in mutant embryos, we performed immunostaining with MVH (mouse vasa homolog), a known germ-cell-specific marker [19]. MVH staining was readily apparent in ovaries from both wild-type controls and *Gcnf*-mutant embryos (Fig. 4B). In *Gcnf*-mutant ovaries, germ cells appeared to be of normal number and morphology as compared to wild-type ovaries.

### *Gcnf* is not required for repression of *Oct4* and *Nanog* in fetal ovarian germ cells

**Figure 5:**
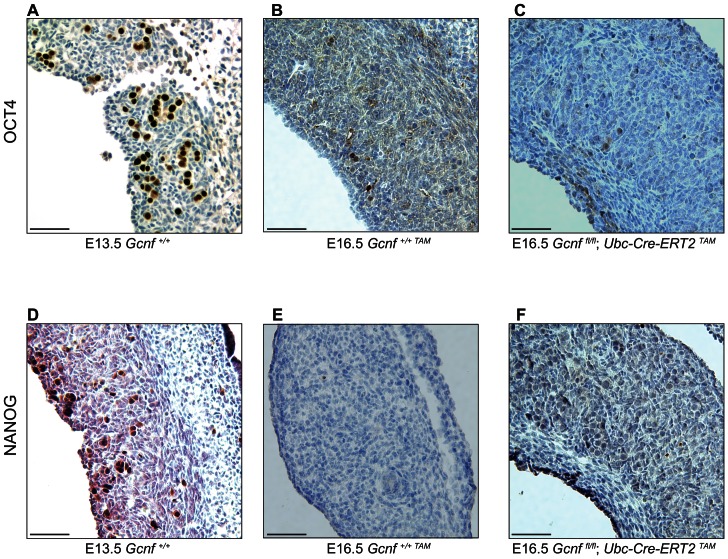
*Gcnf*-mutant germ cells down-regulate OCT4 and NANOG similarly to wild type. Immunostaining for OCT4 (A, B, and C) and NANOG (D, E, and F) proteins in ovary sections. At E13.5 OCT4 and NANOG are readily detectable in *Gcnf^+/+^* wild-type ovarian germ cells (A and D, respectively). At E16.5, as expected, OCT4 and NANOG have been down-regulated and are undetectable in *Gcnf^+/+TAM^* wild-type germ cells (B and E). E16.5 *Gcnf^fl/fl^;Ubc-Cre-ERT2^TAM^* mutant germ cells do not express OCT4 and NANOG and are indistinguishable from wild type (C and F). Anterior is to the left. Scale bar, 50 um.

Because *Gcnf* is required for the repression of *Oct4* and *Nanog* during gastrulation [10], [11], we hypothesized that it might also be required for their repression in fetal ovarian germ cells. If this were the case, *Gcnf*-deficient germ cells would continue to express OCT4 and NANOG at E15.5 and E16.5, when these proteins are undetectable in wild-type ovarian germ cells [5], [13]. We used immunostaining to examine OCT4 and NANOG protein in ovaries from *Gcnf*-mutant (*Gcnf^fl/fl^; Ubc-Cre-ERT2^TAM^*) and wild-type control (*Gcnf^+/+TAM^*) littermates. For each embryo, we examined one ovary by immunostaining and extracted RNA from the other to verify by qRT-PCR whether exon 7 of *Gcnf* was efficiently deleted. As expected, both OCT4 and NANOG proteins were readily detectable in *Gcnf^+/+^* wild-type ovarian germ cells at E13.5 (Fig. 5A,D), but neither was detectable at E16.5 in *Gcnf^+/+TAM^* wild-type ovaries (Fig. 5B,E). *Gcnf*-mutant ovaries were indistinguishable from those of wild-type littermates, with neither OCT4 nor NANOG protein detectable at E16.5 (Fig. 5C,F), indicating that both genes are down-regulated appropriately in the absence of GCNF (Fig. 5). Similar results were observed at E15.5 (data not shown). We conclude that *Gcnf* is not required for the proper down-regulation of OCT4 and NANOG in fetal ovarian germ cells.

### *Gcnf* is not required for meiotic initiation or early oogenesis

**Figure 6:**
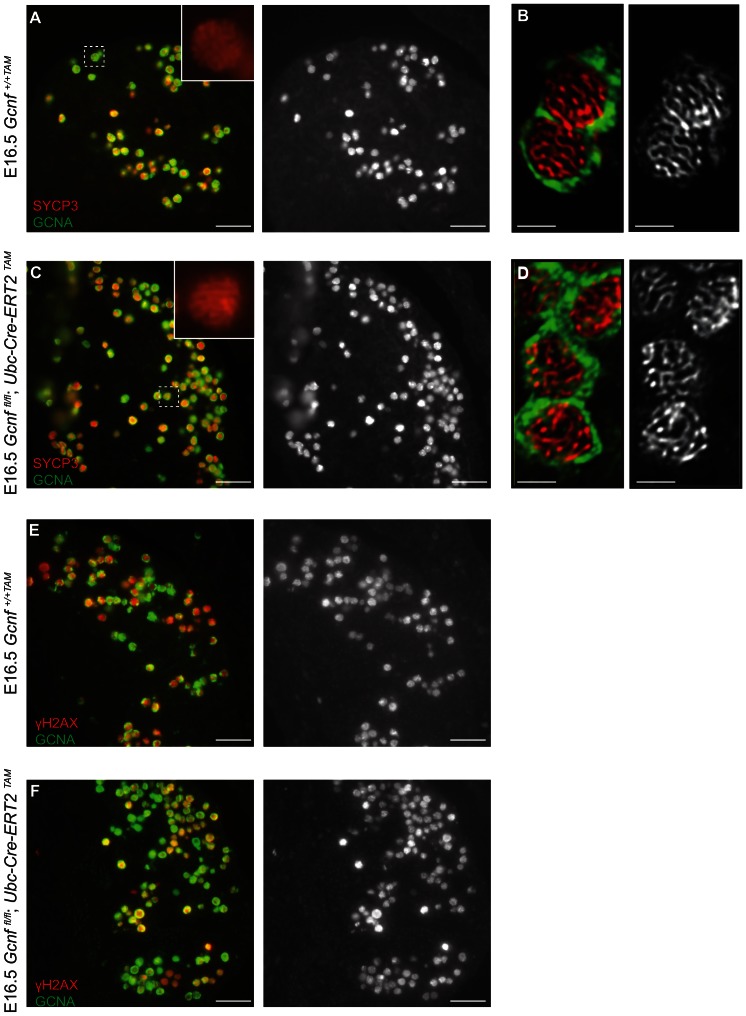
Gcnf mutants undergo meiosis normally. (A–D) Immunofluorescence for SYCP3 (red) and GCNA (green) in *Gcnf^+/+TAM^* wild-type (A,B) and *Gcnf^fl/fl^;Ubc-Cre-ERT2^TAM^* mutant (C,D) ovaries at E16.5; insets show higher magnification of areas boxed in white, SYCP3 staining alone. 100% of GCNA+ cells in both wild type (60/60) and mutant (84/84) were also SYCP3+. B and D, Deconvolution microscopy images demonstrating thread-like staining of SYCP3. Single-channel images showing SYCP3 alone are shown to the right of two-channel images. (E and F) Immunofluorescence for γH2AX (red) and GCNA (green) in *Gcnf^+/+TAM^* wild-type and *Gcnf^fl/fl^;Ubc-Cre-ERT2^TAM^* mutant ovaries at E16.5. Single-channel images are shown to the right. Anterior is to the left. Scale bars, 50 um (A,C,E,F) or 5 um (B,D).

In light of this negative result, we next considered whether other aspects of germ cell development were affected in *Gcnf*-mutant ovaries, namely meiotic initiation and oogenesis. We began by examining expression of meiotic markers. SYCP3 (synaptonemal complex protein 3) is part of the lateral element of the synaptonemal complex, which forms between homologous chromosome pairs during meiosis. Phosphorylated histone H2AX (γH2AX) marks sites of DNA double-strand breaks that form during meiotic prophase. Both SYCP3 and γH2AX are readily detectable in wild-type ovarian germ cells at E16.5 [20]. We used immunostaining to examine wild-type control (*Gcnf^+/+TAM^*) and *Gcnf*-mutant (*Gcnf^fl/fl^; Ubc-Cre-ERT2^TAM^*) ovaries for SYCP3 and γH2AX at E16.5, using the germ-cell marker GCNA (germ cell nuclear antigen) to identify germ cells (Fig. 6). In *Gcnf*-mutant as well as wild-type control ovaries, SYCP3 was expressed in the nuclei of all germ cells (Fig. 6A–D). Higher magnification revealed the threadlike structures of condensed chromosomes (Fig. 6B,D). γH2AX was also found in all germ cell nuclei of *Gcnf*-mutant as well as wild-type control ovaries (Fig. 6E,F). *Gcnf*-mutant and wild-type ovarian germ cells were indistinguishable from each other in staining and structure. These results indicate that meiotic initiation and early meiotic prophase proceed normally in ovarian germ cells lacking GCNF.

**Figure 7:**
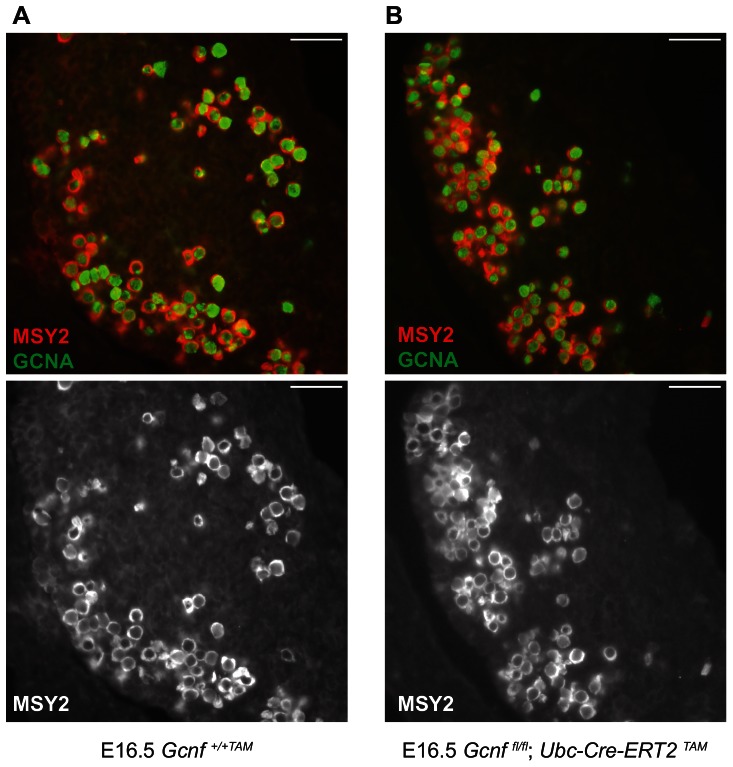
Gcnf mutants initiate oogenesis. Immunostaining for MSY2 (red) and GCNA (green) in *Gcnf^+/+TAM^* wild-type (A) and *Gcnf^fl/fl^;Ubc-Cre-ERT2^TAM^* mutant (B) ovaries at E16.5. Anterior is to the left. Scale bar, 50 um.

Lastly, we tested whether oogenesis was affected in *Gcnf* mutants. The Y-box protein MSY2 (also called YBX2) is expressed in the cytoplasm of maturing oocytes during the diplotene stage of meiosis I [21], [22]. We assayed for MSY2 expression in wild-type and *Gcnf*-mutant ovaries at E16.5 by immunofluorescence, identifying germ cells by expression of GCNA. All germ cells were positive for MSY2 in *Gcnf*-mutant and wild-type ovaries (Fig. 7), indicating that early oogenesis is unaffected by the absence of *Gcnf*. Taken together, these results indicate that *Gcnf* is not required for the early steps of meiosis and oogenesis.

## Discussion

The repression of pluripotency genes in ovarian germ cells around the time of meiotic initiation is a significant aspect of their development, and yet very little is known about this event. *Dazl* (*Deleted in azoospermia-like*) has been shown to be required for primordial germ cells to differentiate following their migration to the gonads. Subsequent downstream events, including down-regulation of pluripotency markers, are disrupted in the absence of *Dazl* [9]. Because *Dazl* controls a broad differentiation event several days before pluripotency marker repression, this is likely an indirect effect of *Dazl* function. We set out to determine what molecular regulators might mediate *Dazl*'s effects in the repression of *Oct4* and *Nanog*.

*Gcnf* represented a good candidate for this role because it is a known direct repressor of *Oct4* and *Nanog* in somatic cells, both *in vivo* and *in vitro* [10], [11]. Here we showed that *Gcnf* is expressed in ovarian germ cells at about the time that *Oct4* and *Nanog* are down-regulated in these cells. However, when we conditionally disrupted the LBD of *Gcnf* in the fetus, we found that, despite its role in *Oct4* repression during gastrulation, *Gcnf* is not required for the regulation of *Oct4* or *Nanog* in ovarian germ cells. In addition, *Gcnf* appears to be dispensable for the initiation of meiosis and oogenesis in fetal ovarian germ cells.

We have considered and ruled out several alternative explanations for our findings. One formal possibility is that ablation of *Gcnf* function was inefficient with our conditional allele, but the available evidence does not support this explanation. Each embryo provided a pair of ovaries, of which one ovary was used for RNA extraction and RT-PCR, while the other was processed for paraffin sectioning and immunostaining. Quantitative RT-PCR on individual mutant ovaries showed an average of less than 1.5% of exon 7 transcript remaining, indicating that exon 7 was deleted in the vast majority of germ cells.

We demonstrated that, in wild-type embryos, *Gcnf* transcript is expressed in germ cells and somatic cells at E9.5, becomes nearly undetectable in both tissue types at E10.5, and is expressed specifically in germ cells at E14.5. Because we induced the exon 7 deletion at E10.5, it is theoretically possible that small amounts of intact GCNF protein expressed at E9.5 persisted in germ cells until E14.5, when *Oct4* and *Nanog* are down-regulated. However, the germ cell population expands from about 1,000 cells at E10.5 to more than 25,000 cells at E13.5 [23], which would have severely diluted any remaining GCNF protein. Therefore, it seems unlikely that enough GCNF protein would remain in every germ cell to effectively carry out its function.

Another possibility is that the DNA binding domain (DBD) is translated and functional in the absence of the ligand-binding domain (LBD). Given the modular structure of nuclear receptors, including GCNF, it is quite possible that the N-terminal portion of the protein, including the DBD, is produced and properly folded. Unfortunately, we were unable to examine whether the DBD was present in ovarian germ cells due to the lack of appropriate antibodies. However, GCNF has been shown to interact with SMRT and NCoR, two well-characterized nuclear co-repressors [11], [12]. Both of these co-repressors interact specifically with the LBD of GCNF, which is typical of their interactions with other nuclear receptors as well. In addition, we demonstrated that the LBD is required for GCNF function earlier in somatic development, and that in its absence mutants display a phenotype remarkably similar to *Gcnf* mutants lacking the DBD. Both mutants demonstrate a failure to turn from the lordotic to fetal position, a failure to completely close the neural tube, and truncated somitogenesis, as well as highly elevated *Oct4* expression at E9.5 [11], [15], [16]. Although some of these defects can be observed in other mutants, taken together they suggest that disruption of the LBD does in fact abrogate GCNF function and that the LBD is required during *Gcnf's* role in earlier development, when *Gcnf* is thought to be required for posterior patterning [15]. Therefore, although we cannot formally rule out the possibility, it is unlikely that the DBD represses *Oct4* and *Nanog* in the absence of the LBD during female germ cell development.

A more plausible explanation for our findings is that *Oct4* and *Nanog* are regulated by different enhancers in the germline and the gastrulating embryonic soma. Indeed, there is published evidence of such enhancer-controlled tissue specificity in the case of *Oct4*. Yeom and colleagues demonstrated that there are two functionally separable enhancer elements present in the *Oct4* promoter, one of which is more proximal to and the other more distal to the transcriptional start site [24]. The proximal enhancer (PE) drives expression of *Oct4* in the epiblast and epiblast-derived cells such as embryonal carcinoma cells. The PE is required for expression of *Oct4* in the cells of the epiblast around the time of gastrulation. However, the distal enhancer (DE) is sufficient for expression of *Oct4* in the pre-implantation embryo as well as in the germline, even in the absence of the PE.

It is unclear how these enhancer elements are differentially regulated, but it seems likely that different transcription factors bind to the two enhancers. GCNF is thought to perform its repressive function by competing with activating nuclear receptors for binding sites in promoters, and it has been shown to bind to response elements in the proximal promoter of *Oct4* near, though not within, the PE [11], [25]. It is possible that, during gastrulation, GCNF's proximity to the PE disrupts the activity of transcriptional activators that normally bind there, resulting in the GCNF-dependent repression of *Oct4* observed in gastrulating embryos and ES cells [11]. In germ cells, on the other hand, GCNF may bind to the proximal promoter but may not be sufficient to repress *Oct4* due to its distance from the DE. In this case, other factors, perhaps binding in or near the DE, would be responsible for repressing *Oct4* in the germline.

Our experiments indicate that, although the LBD of *Gcnf* is required for repression of *Oct4* and *Nanog* in somatic cells, it is not required for repression of these pluripotency factors in ovarian germ cells. The finding that pluripotency genes are differentially regulated in somatic and germ cells is intriguing, and it will be interesting to understand what factors contribute to this disparity. It may be due to differential regulation of enhancer elements in the promoters of these genes in somatic versus germ cells. Additionally, we cannot rule out the possibility that other factors act redundantly or in conjunction with *Gcnf* in this context. Finally, it will be important to determine what factors mediate *Dazl*'s effects in the repression of pluripotency factors in fetal ovarian germ cells, and whether this repression of pluripotency factors is required for the initiation of meiosis and oogenesis.

## Materials and Methods

### Ethics statement

All experiments involving mice conformed to ethical principles and guidelines approved by the Committee on Animal Care at the Massachusetts Institute of Technology (IACUC number: 0711-075-14).

### High throughput mRNA sequencing

The *Gcnf* expression data shown in Fig. 1A were kindly provided by Jacob Mueller and Mark Gill, who are conducting an analysis of fetal gonadal gene expression (personal communication). In brief, total RNA from E12.5, E14.5, and E16.5 gonads from wild-type and *W/W^v^* embryos was processed using the GLOBINclear Kit (Ambion, AM1981) to remove hemoglobin mRNA. cDNA libraries for sequencing were prepared using the TruSeq RNA Sample Prep kit (Illumina). 36-base-pair reads were sequenced on an Illumina GAII sequencer. Reads were aligned to *Mus musculus* genomic sequence (mm9) using Tophat [26] and reads-per-million were estimated using custom Perl script (available upon request).

### Single molecule fluorescence in situ hybridization

Fetal urogenital ridges were dissected and fixed overnight in 4% paraformaldehyde in PBS, then equilibrated in 30% sucrose, 4% paraformaldehyde in PBS before being frozen in OCT compound (Tissue-Tek). Eight-micron sections were hybridized as described in [27], using AlexaFluor 488-conjugated anti-SSEA1 (BD Pharmigen, BD560172) to label germ cells. The DNA probes used to visualize *Gcnf* transcript were conjugated to Cy-5, and their sequences were as follows (all listed 5′ to 3′): TCATCCTGACAGAAACCGTT; ATTAGTGCCTGGATCAAGCT; GCCAAGTGTTAAACTGTCAG; TGGGACGGAAACAGGTATAT; TTCGTTGTTCAGCTCGATCA; AGATGATCCCATAGTGCAAG; TCTTGAAAAACCCCTTGCAG; ACCCGTTTGTTGCAAATGCT; ACACAGTTCTTGTCACGACT; TGACATCTGTTCCTCTGCTT; ATCTGGAGACACTTGAGCAG; TGATAGCCTTCCTGTTCATG; ACTGGTCCAATGCTCTTGTT; CCTGTCCAGACATGATTCTT; GTGATTGGCTTCTTCCTCAA; TTGCTCTCTGAAGCCCTGTT; ATGATAGTGTGGAGCCTGGT; CCATTTAGTTCCACAGACCT; TACTGATCCCTGAATGCCAT; TAATGTGGAGGCACTGACAT; GCTAAAAAGGTGTGGTATGT; AAAGTGGTGAGTGGCCAGAA; ATCAGACTGTAGGACTGAGG; TTCGGCTGACATCAGCTGAT; TCAACATAGGTGTGCCCAAT; TGTCACAGCATACCCATCTT; AAGCAGAGCAAACAGTTCTG; TGCCTAAAGAGCAACTCGTC; CAGCTTCTTGATCCAGGCAA; TTGAGAGCTCGCAGAAGAAA; AAGAGGCACGTGTAATCCTT; ATTAACTCCTGCCACGTAGA; ATCTGCTTGCTGTACACTGT; AGTACTTGGCTGTGACATCA; TGTGGAGTTCTTCATCAGAG; CTCCATCCCTTCATCACTAA; GGTAGATGAGTCGTTCAATC; CAGCTGATGGAACTTGTGAT; TTTCATGCATGCGTACTCCT; CCCTGATATCTTGATTCAGG; TTCAGTTGTTCCAGCTGTGA; GACAAATGTACCAATACCGC; GGTTTGGCTGATGTGTGTAT; GCACATCATAAGATCAGGAA; TGCGATGTATCGGATCTCTG; AGGGGCACATTCACCATCTT; AGCACCACCTTAAAGAGGAG; TTCACCGTACTTGTCTTGCA.

### Mice

All studies were performed on an inbred C57BL/6 background. C57BL/6 *Gcnf^gt/+^* targeted ES cells were obtained from the EUCOMM repository. The sequence of the targeting construct is available at: http://www.knockoutmouse.org/targ_rep/alleles/4557/escell-clone-genbank-file. ES cell clones were injected into Balb/c blastocysts and transferred to pseudopregnant CD1 females to generate chimeras. Animals were genotyped by PCR using primers that amplify the region containing the 3′ LoxP site (fwd: TGCAAGGCCAGGTCTTTAAC; rev: AGGAGCCCTTCCAAGTTACC). *Gcnf^fl/+^* animals were generated by mating *Gcnf^gt/+^* heterozygotes to mice carrying *ACTB:FLPe* [17] to delete the gene trap and neomycin resistance cassette. Offspring were genotyped by PCR for the 3′ LoxP site as described above, as well as with primers that flank the region containing the gene trap and neomycin resistance cassette (fwd: TGGGGCACTGTAACAAGACC; rev: TGACAATCTCCTACTTGCCCTACC). Primers for detecting the presence of *Ubc-Cre-ERT2* were as follows: fwd: GATAGTGAAACAGGGGCAATGGTGC; rev: TAGAGTATGGGGGGCTCAGCATCC. *Gcnf^+/+^*, *Gcnf^Δ/+^*, and *Gcnf^Δ/Δ^* embryos were distinguished using forward primers in exon 7 (TGGAGCAACCATGGTGACAG) or overlapping the 3′ loxP site (ACGAAGTTATGGTCTGAGCTC), together with a common reverse primer downstream of the loxP site (GAGCCCTTCCAAGTTACCTC).

### *Gcnf*-mutant embryos

To obtain *Gcnf^Δ/Δ^* mutant embryos, *Gcnf^Δ/+^* heterozygous males and females were mated. To establish timed matings, female mice were housed with male mice overnight. Noon of the day when a vaginal plug was evident was considered E0.5. Embryos were dissected into PBS at E9.5, and yolk sacs were reserved for genotyping. To obtain *Gcnf^fl/fl^; Ubc-Cre-ERT2* mutants, *Gcnf^fl/+^* females were mated to *Gcnf ^fl/+^; Ubc-Cre-ERT2* males. Embryonic gonads and mesonephroi were dissected into PBS at E15.5 and E16.5, with tail samples reserved for genotyping. The sexual identity of the gonads was determined by scoring the presence or absence of testicular cords.

### Tamoxifen administration

To induce recombination of the *Gcnf^fl^* allele, tamoxifen (Sigma) was dissolved at 20 mg/mL in corn oil, and administered to adult males or pregnant females via intraperitoneal injection at 5 mg tamoxifen/40 g mouse. Each pregnant female was injected twice, at E10.5 and E11.5. Adult males were injected once per day for four consecutive days.

### Immunostaining

Fetal ovaries were fixed overnight at 4°C in 4% paraformaldehyde in PBS, embedded in paraffin, and sectioned. Slides were dewaxed, rehydrated, heated in Antigen Retrieval Buffer 1 (Spring Bioscience) for 8 min, and blocked for 30 min in 2.5% horse serum (colorimetric) or 3% goat serum (fluorescence). Slides were then incubated with primary antibody for 1 h, diluted as follows: anti-NANOG (Bethyl Laboratories, IHC-00205) 1∶200; anti-OCT4 (BD Transduction Labs, 611203) 1∶100; anti-MVH (Abcam, ab13840): 1∶250; anti-SYCP3 (Santa Cruz, sc-74569) 1∶200; anti-γH2AX (Millipore, 05-636) 1∶200; anti-MSY2 (a gift from Richard Schultz, University of Pennsylvania) 1∶200; anti-GCNA (a gift from George Enders, University of Kansas) undiluted. For colorimetric detection, slides were incubated with rabbit ImmPress reagent (Vector Labs) and developed using ImmPACT DAB substrate (Vector Labs), then counterstained with hematoxylin, dehydrated, and mounted using Permount (Fisher Scientific). For fluorescence detection, slides were incubated with FITC-conjugated anti-rat and Rhodamine-conjugated anti-rabbit or anti-mouse (Jackson ImmunoResearch Laboratories) at 1∶250 dilution, then mounted with VECTASHIELD mounting media with DAPI (Vector Labs). Deconvolved images of SYCP3 immunostaining were obtained using a DeltaVision Elite imaging system (GE Healthcare).

### Quantitative RT-PCR

To examine *Oct4* expression in *Gcnf^Δ/Δ^* and wild-type embryos, quantitative RT-PCR (qRT-PCR) was performed on whole embryo samples. To examine *Gcnf* expression in *Gcnf^fl/fl^; Ubc-Cre-ERT2^TAM^* and wild-type ovaries, qRT-PCR was performed on ovaries dissected away from the mesonephroi. In both cases, samples were submerged in TRIzol reagent (Invitrogen) and stored at ^−^80°C. After genotyping of the yolk sac or tail, total RNA was prepared according to the manufacturer's instructions. cDNA was transcribed from 100–200 ng of RNA using SuperScript III (Invitrogen) according to the manufacturer's instructions. Quantitative PCR was performed on cDNA using SYBR Green Core PCR reagents (Applied Biosystems) on an ABI9700 fast real-time PCR machine (Applied Biosystems). Results were analyzed using the delta-delta Ct method with *Hprt* (hypoxanthine-guanine phosphoribosyltransferase) or *Actb* (actin, beta) as a normalization control. RT-PCR primers were as follows:

*Hprt*: (F) TCAGTCAACGGGGGACATAAA; (R) GGGGCTGTACTGCTTAACCAG

*Actb*: (F) GAAATCGTGCGTGACATCAAAG; (R) TGTAGTTTCATGGATGCCACAG

*Oct4*: (F) CAGCCAGACCACCATCTGTC; (R) GTCTCCGATTTGCATATCTCCTG

*Gcnf exon 7–8*: (F) CACCAGGCTCCACACTATCA; (R) GATCCCTGAATGCCATGAAT
